# Machine Learning-Based Diagnosis in Laser Resonance Frequency Analysis for Implant Stability of Orthopedic Pedicle Screws

**DOI:** 10.3390/s21227553

**Published:** 2021-11-13

**Authors:** Katsuhiro Mikami, Mitsutaka Nemoto, Takeo Nagura, Masaya Nakamura, Morio Matsumoto, Daisuke Nakashima

**Affiliations:** 1 Faculty of Biology-Oriented Science and Technology, Kindai University, Wakayama 649-6493, Japan; nemoto@waka.kindai.ac.jp; 2 Department of Orthopedic Surgery, Keio University School of Medicine, Tokyo 160-8582, Japan; nagura@keio.jp (T.N.); masa@keio.jp (M.N.); morio@keio.jp (M.M.); nakashima@keio.jp (D.N.); 3 Department of Clinical Biomechanics, Keio University School of Medicine, Tokyo 160-8582, Japan

**Keywords:** orthopedics, pedicle screw, stability diagnosis, laser resonance frequency analysis

## Abstract

Evaluation of the initial stability of implants is essential to reduce the number of implant failures of pedicle screws after orthopedic surgeries. Laser resonance frequency analysis (L-RFA) has been recently proposed as a viable diagnostic scheme in this regard. In a previous study, L-RFA was used to demonstrate the diagnosis of implant stability of monoaxial screws with a fixed head. However, polyaxial screws with movable heads are also frequently used in practice. In this paper, we clarify the characteristics of the laser-induced vibrational spectra of polyaxial screws which are required for making L-RFA diagnoses of implant stability. In addition, a novel analysis scheme of a vibrational spectrum using L-RFA based on machine learning is demonstrated and proposed. The proposed machine learning-based diagnosis method demonstrates a highly accurate prediction of implant stability (peak torque) for polyaxial pedicle screws. This achievement will contribute an important analytical method for implant stability diagnosis using L-RFA for implants with moving parts and shapes used in various clinical situations.

## 1. Introduction

With an aging population, the surgical use of orthopedic implants, such as pedicle screws [1,2] and acetabular cups, has increased. In spine surgery, implant failure within six months has become a serious problem, with an incidence as high as approximately 12% [3] and a reported reoperation rate of 40% [4]. In this regard, evaluation of the initial fixation of the implant (implant stability) as a factor that contributes to loosening of screws is essential to prevent implant failure [5,6]. Currently, parameters such as the pull-out force [7] and insertion torque [5,8] are used to evaluate implant stability. The pull-out force test has the limitation of being a destructive laboratory-based test that cannot be used in clinical practice. In contrast, insertion torque can be measured clinically, but only once at the time of insertion, and is affected by subjective factors attributable to each surgeon, such as the speed of insertion and the amount of force applied in the direction of insertion.

Recently, laser resonance frequency analysis (L-RFA) has been demonstrated as a viable method for evaluating implant stability for pedicle screws [9,10] and acetabular cups [11]. L-RFA was developed to replace hammer testing for civil structures using remote laser sensing technology [12,13]. In a hammer test, the condition of an evaluation sample is determined using the vibrations produced by active excitation and by an inspector’s judgement based on auditory perception of the sound propagated through air. In tunnel inspection, the hammer test is used to inspect for defects inside the concrete wall and to find loose anchorage bolts. L-RFA involves inducing vibration by laser ablation or thermoelastic waves [14], and measuring and analyzing the induced vibration using a laser Doppler vibrometer or an acceleration sensor to realize high-speed, remote, and quantitative inspection. Photoacoustic imaging (PAI) using ultrasonic waves generated by laser pulse irradiation has been applied in diagnostic applications, and various developments in this regard have been carried out globally [15]. However, L-RFA focuses on vibrations in audible sound frequency that exist as natural frequencies of evaluated materials. Although laser Doppler vibrometry is relatively expensive, wearable blood flow meters have been reported [16] in recent years, and the technology is expected to be adopted more widely in future.

A diagnosis scheme using RFA frequency was proposed in 1932 [17] and has been studied by several researchers. To date, RFA without laser technology has only been applied medically to dental implants; Osstell ISQ (Osstell) has been used as RFA for dental implants by vibrating the implant with magnetic pulses [18,19,20,21,22,23]. The results are displayed as a unique parameter, the Implant Stability Quotient (ISQ), which ranges from 0 (lowest level of stability) to 100 (highest level of stability). The measured frequency is about 3000 Hz with an ISQ value of 0 and about 8000 Hz with an ISQ value of 100 [20]. There are several laboratory and in vivo studies of noninvasive measurement of implant stability in orthopedics [24,25,26,27,28,29,30,31]. Despite many studies, no methods have been available for use in clinical orthopedic practice. These conventional methods have limitations, requiring complex procedures and huge systems involving vibrators and accelerometers, and could not be used clinically as quantitative methods.

Previous studies on L-RFA in orthopedic implants have evaluated the correlation between implant stability and vibration frequency, which is defined by the highest peak in the vibration spectrum obtained by fast Fourier transform (FFT) analysis (peak frequency). In the index of implant stability, the insertion torque was used for pedicle screws and the pull-down force was used for acetabular cups. The peak frequency refers to a natural frequency, which is determined by the geometry, Young’s modulus, and density of the evaluated sample. It has been found that the peak frequency obtained by L-RFA increases with increasing implant stability [9,10,11]. This demonstration is a milestone in the diagnosis of orthopedic implant stability. However, the pedicle screws in the previous studies were of a monoaxial type, in which the pedicle screw has no moving parts, and there was no mention of polyaxial pedicle screws with a movable head part. Polyaxial pedicle screws are also frequently used in clinical settings. The movable head part of the polyaxial pedicle screw influences natural frequency mode and frequency. Therefore, for the clinical introduction of L-RFA, it is essential to determine the influence of the movable head part.

In this paper, we evaluated L-RFA diagnosis for polyaxial screws to reveal the characteristics of laser-induced vibration. From the obtained results, we clarified the complex vibration specific to polyaxial screws and evaluated the correlation between the conventional peak frequency and insertion torque. A novel machine learning-based analysis scheme for highly accurate diagnosis of implant stability is proposed. The analysis result was predicted implant stability of polyaxial screw, which has not been verified before, and this is demonstrated to have a high accuracy.

## 2. L-RFA Testing for Polyaxial Pedicle Screw

In Section 2, we characterize the results of L-RFA evaluation of polyaxial pedicle screws. Section 1 describes the details of an experiment for L-RFA. Section 2 shows the characteristics of the vibration spectra affected by the moving head part obtained from the measurement results, and Section 3 clarifies the correlation between the vibration frequency and the insertion torque, which is necessary to diagnose the implant stability.

### 2.1. Experimental Setup and Sample

**Figure 1:**
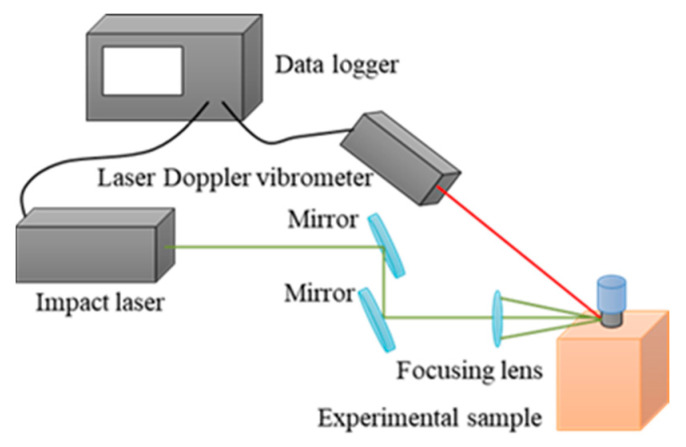
Experimental setup for L-RFA testing.

Figure 1 shows the experimental setup for L-RFA evaluation of polyaxial pedicle screws. A Q-switched Nd:YAG laser with a 1064-nm wavelength and 10-ns pulse width (Dawa-300, Beamtech Optronics Co. Ltd., Beijing, China) was used to induce vibration in an experimental sample. The laser pulse energy was adjusted to 100 mJ by Q-switch timing using a digital delay generator (DG645, Stanford Research Systems Inc., Sunnyvale, CA, USA). The laser pulse was focused using a focusing lens (focal length *f* = 100 mm), and the spot size was 250 μm in diameter, defined as 1/e^2^ values from the peak values of the Gaussian fitting for the beam profile, where e is Napier’s constant. The impact laser pulse was operated at a repetition rate of 10 Hz. A laser Doppler vibrometer (VibroOne, polytec GmbH, Yokohama, Japan) was used as the detection laser for the measurement of the induced vibration. The laser Doppler vibrometer measures the resonant vibrations induced by laser irradiation, which are determined by the mechanical properties and shape of the implant. Therefore, the measured frequency is constant regardless of the measurement direction. On the other hand, the obtained vibration intensity varies depending on the measurement direction. In this study, the effect is neglected by using the same irradiation conditions for all evaluated samples. The laser-induced vibration is very small, on the order of sub-micrometers in displacement. Laser Doppler vibrometers detect vibrations at the focal point of the measurement laser on the order of nanometers and can measure resonant vibrations with high accuracy. In this study, the experimental setup was installed on an optical bread board, and the measurement is not affected by noise originating from the measurement environment. As for the application in the clinical situation, handheld pen-type measurement devices have been discussed in a previous study [9]. The irradiation spot size of the detection laser was approximately 150 μm in diameter. Both lasers were irradiated on the neck of the pedicle screw sample with a 1-mm distance of center for each irradiation laser spot. The vibration signal from the detection laser system was monitored using a data logger (GL7000Plus, Graphtec Co., Yokohama, Japan) with a 0.1-MHz sampling rate and 0.2-Mpoint sampling. This was synchronized with the timing of laser pulse irradiation (Q-switch timing) and data were saved for 2 s, that is, data for 20 pulse irradiations. The obtained signal included laser-induced vibrations with 20 laser pulses divided into individual pulse irradiations and averaged with all divided data. Data acquired until 2 ms after laser irradiation among the analysis data were purged to obtain a clear signal of the low-order natural frequencies. With a short period of impact, a laser pulse width (10 ns) has the potential to induce a broad spectrum of vibrations until ultrasonic vibration (~MHz). Finally, the purged analysis data were analyzed using FFT to obtain a frequency spectrum with a rectangular window function.

**Figure 2:**
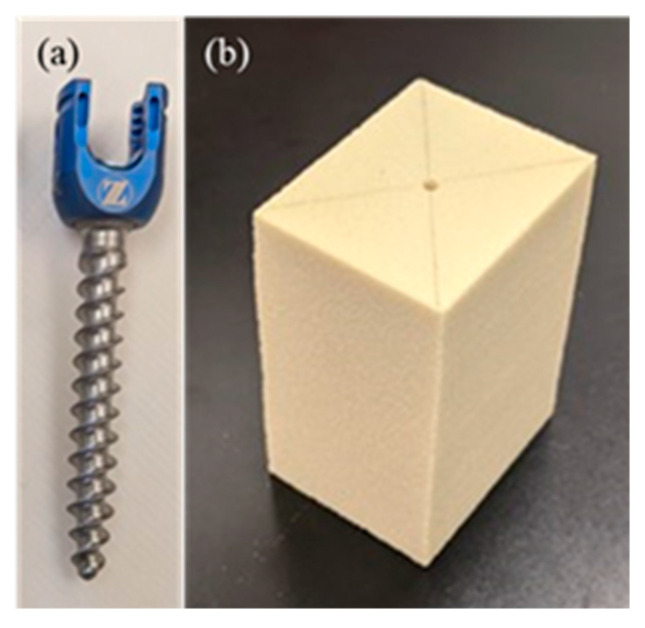
Experimental sample: (**a**) polyaxial pedicle screw, (**b**) artificial bone.

We used polyaxial pedicle screws (701M5535 and 701M6540, Zimmer Biomet) of titanium alloy (Ti-6Al-4V) with lengths of 35 mm and 40 mm and outer threaded diameters of 5.5 mm and 6.5 mm, as shown in Figure 2a. Using an awl, we made a small hole in the target area where an artificial bone was to be inserted at the center of the material. Before insertion, a 2 mm drill bit was used to drill a pilot hole in the material. Then, tapping was performed with a diameter of 1 mm. The pedicle screw was inserted, and the root of the screw was left at 5 mm depth. Insertion of the screw head should be avoided until it comes into contact between the bone and the flange. The screw head causes bone destruction and reduces the fixation force [32]. Therefore, the technique used in our study did not allow the screw head to come into contact with the specimen, which is similar to the situation in practical clinical settings. All screws were inserted at the same depth using a consistent depth gauge. Subsequently, detailed implant stability measurements using insertion torque procedures were performed, as previously described [33]. Four types of solid rigid polyurethane forms (biomechanical test materials, SAWBONES, Pacific Research Laboratory, Inc.) were prepared as biomechanical material to represent the diverse human vertebrae, as shown in Figure 2b. The densities of the artificial bones were 10 pcf (0.16 g/cm^3^), 15 pcf (0.24 g/cm^3^), 20 pcf (0.32 g/cm^3^), and 30 pcf (0.48 g/cm^3^). These densities of the artificial bones can cover human cancellous and cortical bones. They were stacked 40 mm × 35 mm × 60 mm and conformed to the American Society for Testing and Materials (ASTM) standard. In this study, different artificial bones were used to obtain different insertion torques. The densities of the artificial bones used in each of the total 57 times L-RFA measurements were 10, 15, 24, and 26 times for 10, 15, 20, and 30 pcf, respectively. A digital torque gauge (HTGA-5N, IMADA Co. Ltd., Toyohashi, Japan) was used to measure the insertion torque. The specifications of this torque gauge were as follows: accuracy = ±0.5% full-scale  ±  one digit, and sampling rate = 2000 data/s. We measured the insertion torque (Nm) while advancing the screw into the artificial bone, and it was reported to progressively increase with an increase in the number of penetrating screw threads as the screw advanced. Thus, maximum torque was achieved when the screw was inserted to the appropriate length. The surgeon felt the maximum torque to be the strength of the screw fixation, defined as the peak torque [33,34,35]. However, since the torque varies depending on the speed of fastening and the length of implantation (distance between implant head and bone), it depends on the surgeon and is difficult to obtain a stable value.

### 2.2. Influence of Screw Head Mobility of Polyaxial Pedicle Screws

**Figure 3:**
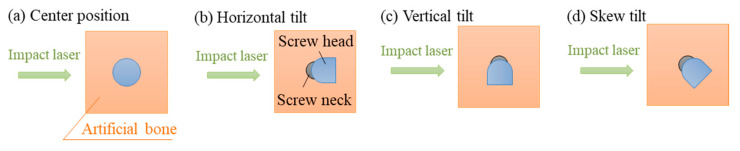
Experimental setting for evaluation of different head directions: (**a**) center position, (**b**) horizontal tilt, (**c**) vertical tilt, and (**d**) skew tilt.

**Figure 4:**
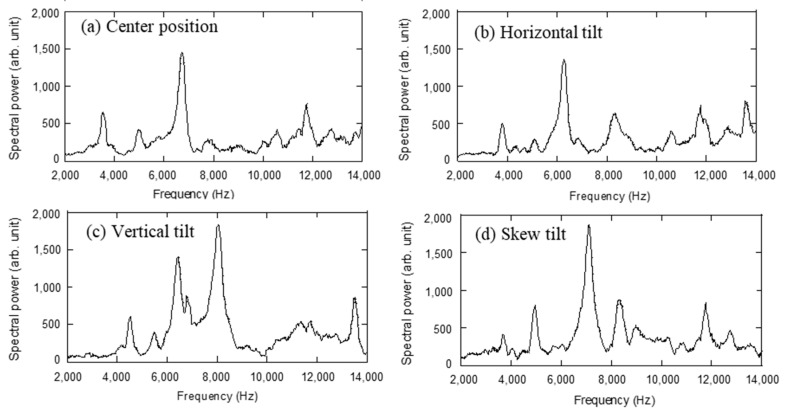
Laser-induced vibration spectra at different tilt directions of screw head: (**a**) center position, (**b**) horizontal tilt, (**c**) vertical tilt, and (**d**) skew tilt.

**Figure 5:**
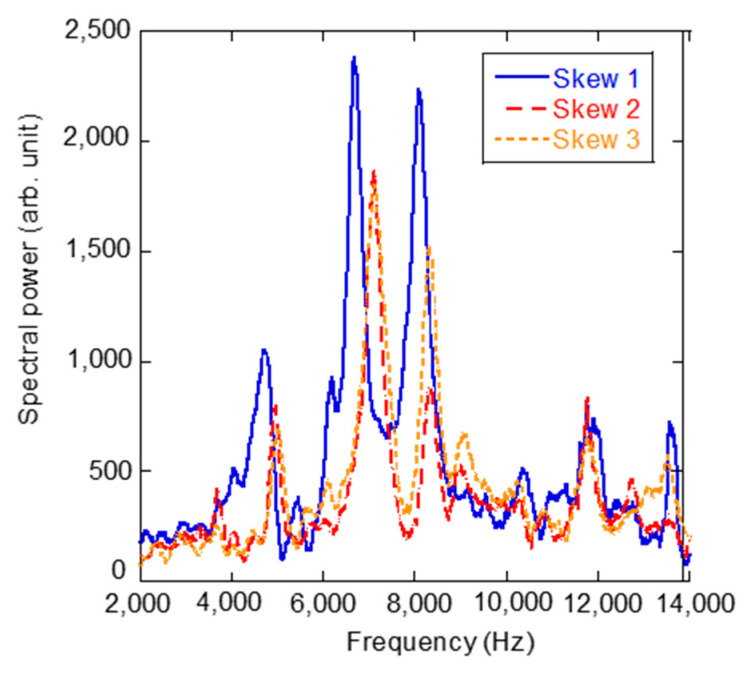
Variation in laser-induced vibration at the same tilt direction when tilted repeatedly.

In order to reveal the influence of the movable head in a polyaxial screw on laser-induced vibration, the head was intentionally tilted in different directions and L-RFA was conducted. Figure 3 shows the tilt directions of the four tilting types of heads: (a) center position, (b) horizontal tilt, (c) vertical tilt, and (d) skew tilt, were each evaluated. We defined the center position as the situation when the movable head stands in the perpendicular direction, the horizontal tilt as the situation when it is tilted coaxially with the laser irradiation direction, the vertical tilt as the situation when it is tilted perpendicular to the laser irradiation direction axis, and the skew tilt as the situation when it is tilted diagonally. Figure 4 shows the vibration frequency spectra of a polyaxial pedicle screw at different head directions, in different tilting types: (a) center position, (b) horizontal tilt, (c) vertical tilt, and (d) skew tilt, with a diameter of 6.5 mm, length of 40 mm, set in a 15 pcf artificial bone at 1.05 Nm insertion torque. When the head was in the center position, a frequency peak appeared at approximately 6740 Hz. When the head was tilted, the peak vibration frequency was decreased for the horizontal tilt and increased for the vertical tilt. In the case of skew tilt, an intermediate trend was indicated between the horizontal and vertical positions. Additionally, as a troubling characteristic of the polyaxial screw, Figure 5 shows the variation in the laser-induced spectra at skew tilt when tilted repeatedly. It is clear from the figure that the L-RFA results do not have the same spectrum at the same skew tilt. These results show that the movable head of the polyaxial pedicle screw affects the laser-induced vibration spectrum. This effect on the spectrum can be attributed to three factors: first, the tilt direction influences the center of gravity of the polyaxial pedicle screw and affects the shape of natural modes. Second, a change in the tilt direction of the head leads to a change in the overall shape of the polyaxial pedicle screw, which causes a change in natural frequency. Third, the shape of the polyaxial pedicle screw head does not have an exact circular head for the placement of a fixation rod. Therefore, it is difficult to obtain a constant tilt condition because of the complex shape. These three factors are difficult to control with the surgical technique making the performance impractical to use L-RFA in clinical settings. In other words, it is essential to obtain an analytical method that can evaluate implant stability from spectra measured at random head orientations of polyaxial pedicle screws.

### 2.3. Relationship between Insertion Torque and Natural Frequency in Polyaxial Pedicle Screws

**Figure 6:**
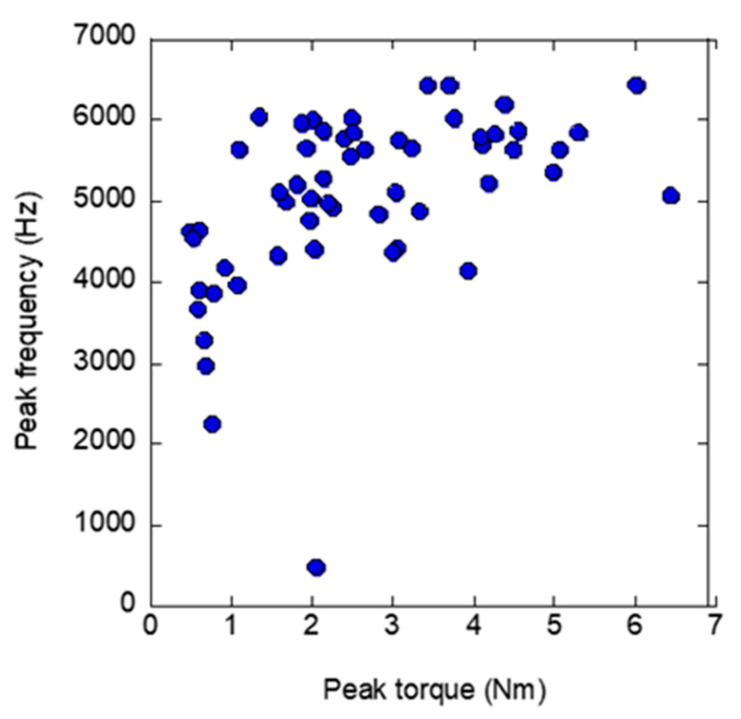
Relationship between peak torque and peak frequency for polyaxial pedicle screws.

Figure 6 shows the results of the correlation between the peak frequency and the peak torque of a polyaxial screw with a diameter of 5.5 mm and a length of 35 mm, with a random tilt direction of the movable head. A similar trend to that of previous studies in which the peak frequency was increased with increasing peak torque was obtained, but the variability in the data was very large. This can be seen from the fact that the coefficient of determination for the logarithmic fitting of the monoaxial screw in the previous study was *R*^2^ = 0.867 [10], while the coefficient of determination for the logarithmic fitting of the results in Figure 6 was lower (*R*^2^ = 0.301). In clinical practice, it is assumed that the results of L-RFA measured intraoperatively will be converted to implantation torque by substituting the pre-determined logarithmic approximation formula. This results in an error of approximately ±1000 Hz even for the same peak implantation torque; furthermore, if the L-RFA results are obtained at 5000 Hz, the implantation torque could range from 0.5 Nm to 6.8 Nm, making it impossible to make a reliable diagnosis.

## 3. Machine Learning-Based Diagnosis for L-RFA

In Section 3, we explain and demonstrate the machine learning-based diagnosis of pedicle screws. Section 1 describes the details of proposed analysis scheme using machine learning. Section 2 shows analysis results with previously experimental result using monoaxial screw to characterize of the proposed scheme, and Section 3 describes the demonstration for the polyaxial pedicle screw described in Section 2.

### 3.1. Analysis Scheme with Machine Learning

**Figure 7:**
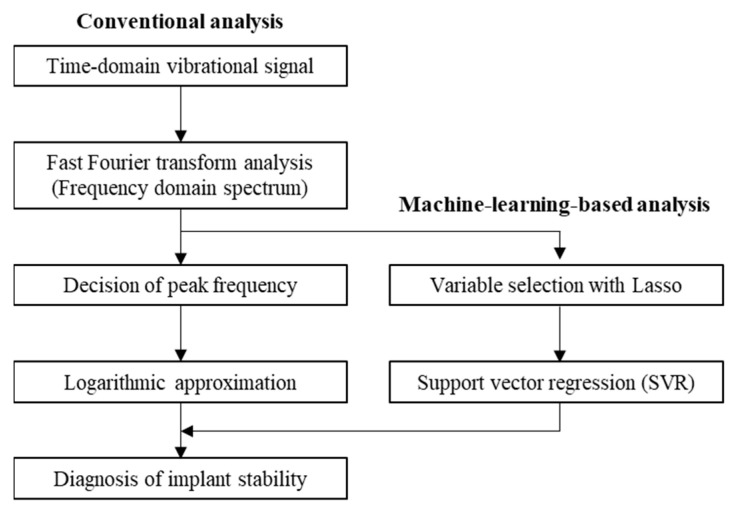
Flowchart of L-RFA analysis scheme for implant stability.

To address the technical difficulty faced with polyaxial pedicle screws in L-RFA, we adapted machine learning to diagnose implant stability, that is, peak torque, which was previously evaluated by focusing only on the peak vibration frequency. Figure 7 shows the installation of the machine learning scheme in the analysis flowchart. In the L-RFA analysis process, the obtained time-domain vibration waveform (i.e., the voltage signal from the laser Doppler vibrometer) is converted into a frequency-domain vibration spectrum via FFT analysis. The frequency with the most potent vibration is extracted from the laser-induced vibration frequency spectrum in the conventional method. Then, the correlation with the insertion torque, which is an implant stability index, is obtained by logarithmic approximation. This study developed an analytical method that can obtain implant stability with high accuracy by introducing a machine learning-based analysis scheme as part of the analytical process. In general, deep learning requires a large amount of high-quality training data. However, insufficient training data is a limiting factor because it is impossible to acquire data repeatedly in a clinical situation. Therefore, it was essential to consider a machine learning scheme that can be expected to have high accuracy even with a small amount of training data.

Our proposed machine learning-based analysis process was constructed using two schemes. The first scheme introduces Lasso [36,37] as an explanatory variable selection method. Previously, the correlation was evaluated by a logarithmic approximation of the peak torque using an individual peak frequency in a limited frequency range in the spectrum. In the vibration frequency spectrum, many explanatory variables, such as its intensity and variance, are spread over the entire measurement frequency band. Therefore, it is possible to divide the frequency band in which the analysis is conducted and extract each explanatory variable for each range. However, a regression estimation with many unnecessary explanatory variables often leads to low regression accuracy and overlearning. It is difficult to select only essential variables from many explanatory variables using manual analysis methods. Lasso is a method used to estimate a sparse linear regression function by the L1 regularization. Lasso adjusts the coefficients of unnecessary explanatory variables to zero to avoid overfitting the estimated function for the training dataset. The use of linear regression in the variable selection is expected to enable the investigation of essential parameters for the basic tendency that the peak torque increases with an increase in certain variable parameters, and this led to the suppression of overlearning. In the proposed method, this Lasso based variable selection is introduced with the bootstrap method. The bootstrap method was used to generate a large number of samples by performing data recovery and extraction, random sampling with replacement, to allow the estimation of the sampling distribution of almost any statistic using random sampling methods. This helps in improving the accuracy of explanatory variable selection. As a specific procedure, normalization was performed to flatten the order of explanatory and objective variables. To determine an adequate regularization parameter, Lasso was performed 2000 times to extract 40 explanatory variables and determine the regularization parameter in the best case of a determination coefficient in Lasso regression. Using the determined regularization strength, the important explanatory variables were ranked by sorting them in the order of the number of times they were selected.

The second scheme uses support vector regression (SVR) for nonlinear regression. SVR is an adaptation of a support vector machine (SVM) [38,39], a pattern recognition method to detect deterioration, and can be adapted to nonlinear problems by incorporating kernel functions. In this study, we used a Gaussian kernel (RBF kernel), which is known to be faster than other kernels in terms of computation time and accuracy. As with Lasso, SVR was normalized to flatten the order of the explanatory and objective variables. The hyperparameters were determined using grid search for training SVR, and the ranges were 2^−20^–2^9^ for the epsilon-insensitivity loss function *ε*, 2^−10^–2^10^ for the regularization constant *C*, and 2^−15^–2^9^ for the RBF kernel function γ. These hyperparameters were obtained in the training case with the highest determination coefficient on three-fold cross-validation. Finally, for the validation of unknown data, the trained SVR was adapted for regression, and the predicted value of the implant stability (peak torque) was presented.

### 3.2. Characterization of Machine Learning-Based Diagnosis Method

**Figure 8:**
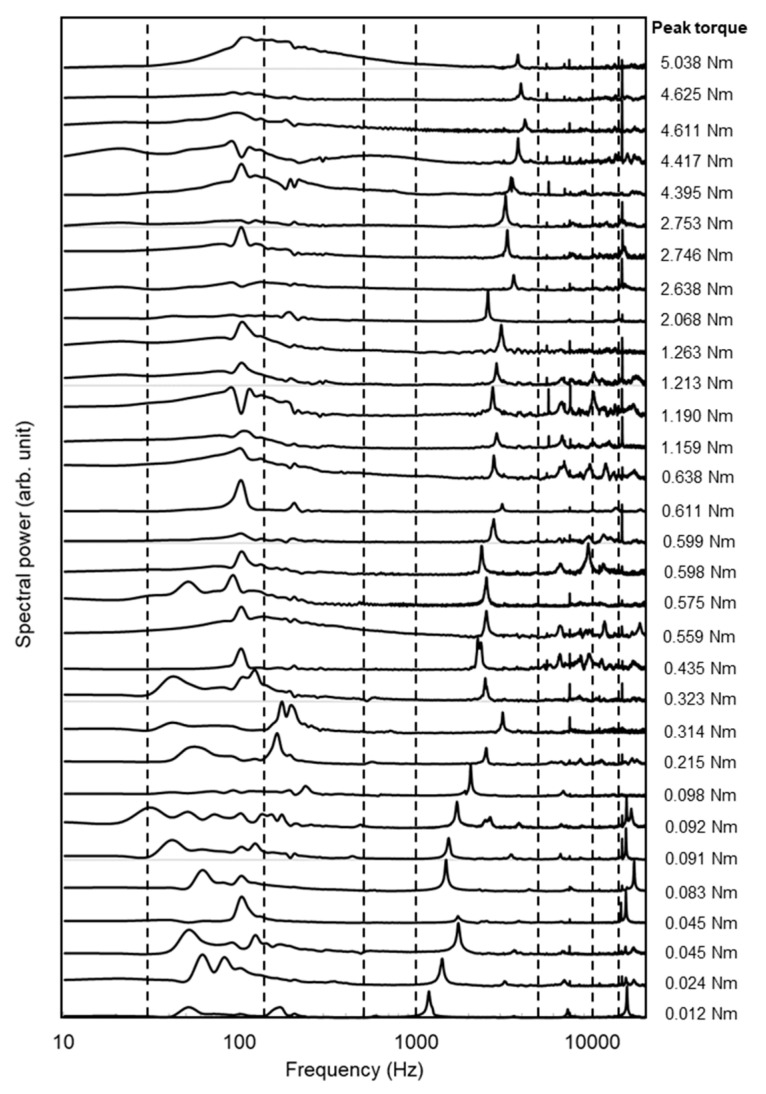
Laser-induced vibration spectra for monoaxial pedicle screws reported in [10]. The dashed lines show analysis ranges for calculations of each explanatory variable.

**Figure 9:**
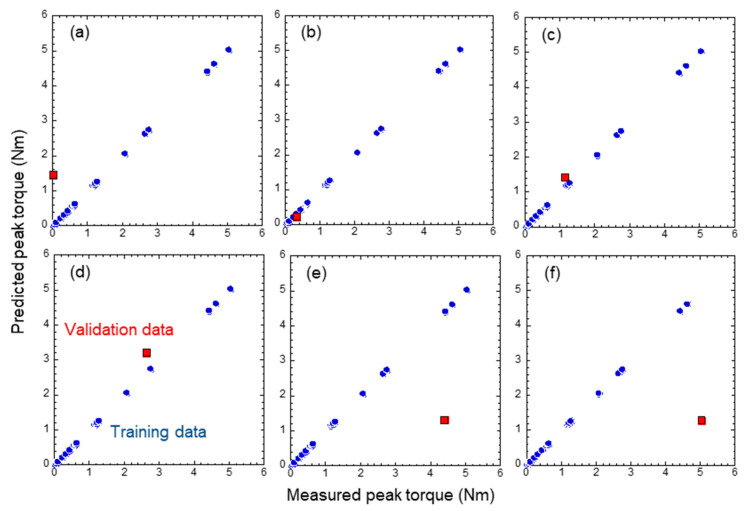
Leave out one cross-validation of machine learning-based diagnosis with L-RFA result for monoaxial pedicle screws reported in a previous study [10]. The blue plot shows the training data, which inevitably outputs exactly the same prediction results as training data, and the red plot shows validation data that is not included in the training data. Figure 9a,f show the analysis results at the minimum and maximum peak torques. Figure 9b–e show the typical analysis results in between. The peak torques of validation data are (**a**) 0.012 Nm, (**b**) 0.323 Nm, (**c**) 1.159 Nm, (**d**) 2.753 Nm, (**e**) 4.395 Nm, and (**f**) 5.038 Nm.

The L-RFA results were evaluated in a previous study [10] for a monoaxial screw (CMS05135, Kyocera Medical Corporation) with a length of 45 mm and a diameter of 5.5 mm placed in an artificial bone and were re-analyzed to clarify the effectiveness of the machine learning-based analysis. Figure 8 shows the laser-induced vibration spectra measured by L-RFA in the previous study [10]. In this study, eight variables (peak frequency, peak intensity, centroid frequency, centroid intensity, average intensity, dispersion, kurtosis, and skewness) were extracted in eight frequency ranges (30–150 Hz, 150–500 Hz, 500–1000 Hz, 1000–5000 Hz, 5000–10,000 Hz, 10,000–15,000 Hz, 15,000–20,000 Hz, 30–20,000 Hz), resulting in a total of 64 explanatory variables. Here, the dispersion *V*, kurtosis *K*, and skewness *S* in each frequency range were calculated with equations:

(1) $$V=\frac{1}{n}\overset{n}{\underset{i=1}{\sum }}{\left(x_{i}-\bar{x}\right)}^{2}$$

(2) $$K=\frac{n\left(n+1\right)}{\left(n-1\right)\left(n-2\right)\left(n-3\right)}\overset{n}{\underset{i=1}{\sum }}\frac{{\left(x_{i}-\bar{x}\right)}^{4}}{s^{4}}-\frac{3{\left(n-1\right)}^{2}}{\left(n-2\right)\left(n-3\right)}$$

(3) $$S=\frac{n}{\left(n-1\right)\left(n-2\right)}\overset{n}{\underset{i=1}{\sum }}{\left(\frac{x_{i}-\bar{x}}{s^{4}}\right)}^{3}$$

where, *n* is the number of data, *x_i_* is the intensity of the *i*th datum, $\bar{x}$ is average intensity, and *s* is standard deviation. The dashed lines in Figure 8 show the frequency range where the variables were extracted, and the divided range was determined manually so that several characteristic peaks could be separated. Table 1 shows the explanatory variables extracted in more than 50% of the samples in trials using Lasso. The regularization parameter was 0.03, the coefficient of determination was 0.289, and the average number of explanatory variables extracted per trial was 8.4. The most frequently extracted variable in the Lasso results was the peak frequency of 1000–5000 Hz, indicating that the same explanatory variable as in previous studies is the most important. The outcome of using these results to verify the performance of the machine learning-based diagnosis by SVR using eight explanatory variables selected by Lasso is shown in Figure 9. Datasets of 31 from the previous study obtained, which were separated into 30 training datasets and one validation dataset left out for cross-validation. The validation dataset was assumed as unknown data to predict the implant stability, that is, the peak torque. This is intended for clinical applications where measured anonymous data are fed into a learning model. The blue plot inevitably outputs exactly the same prediction results as the training data. In contrast, the red plot shows validation data that were not used for training. When the results of the validation data, as shown with the red plot, converged with the linear line defined with equation *y* = *x*, represented by the blue plots, a more accurate prediction was observed. Figure 9 shows the results with different validation data to reveal the characteristics of the proposed machine learning-based diagnosis method. Figure 9a,f show the analysis results at the minimum and maximum peak torques. Figure 9b–e show the typical analysis results in between. From the validation results, two characteristics of the proposed machine learning-based diagnosis were revealed. First, it is sensitive to extrapolated data. The upper left and lower right graphs in Figure 9 show the results when the lowest and highest peak torque results are used as the validation data. It can be seen that the prediction accuracy of the unknown data outside the training data range is significantly inferior. Second, a homogeneous and large amount of training data is required. The bottom center graph shows that the prediction accuracy decreased in areas where the amount of training data was small, and the plot is sparse, although it was not extrapolated data. In contrast, as shown in the upper center graph, the prediction accuracy was extremely high when the plots were dense with a large amount of training data. From Figure 9b–d, it is possible to predict the results with accuracy within a 20% margin of error in the range of 0.06 Nm to 3.00 Nm. This indicates that we are able to measure the ambiguous region of implant stability that cannot be judged by a surgeon in clinical practice as being too weak or strong enough. The fastening torque of 3 Nm is the standard for M5 bolts specified in Japanese Industrial Standards. Therefore, since the torque required for industrial bolts has been obtained, it can be considered that the torque has been measured to a sufficient level.

### 3.3. Demonstration of Diagnosis for Polyaxial Pedicle Screw

The proposed method was subsequently adapted to polyaxial screws, which are difficult to assess using conventional analysis schemes. Since the resonant frequency is determined by the mechanical properties and shape, the vibration spectrum to be measured by L-RFA depends on the implant. Therefore, the frequency ranges used in machine learning-based analysis need to be optimized for each implant. To perform Lasso, the analysis frequency range was defined manually from the frequency spectrum obtained by L-RFA in six frequency bands for the polyaxial screw: 0–1500 Hz, 1500–6500 Hz, 6500–11,500 Hz, 11,500–15,000 Hz, 15,000–25,000 Hz, and 0–25,000 Hz. The same eight variables as for the monoaxial screw were used; thus, a total of 48 explanatory variables were prepared. The explanatory variables extracted more than 50% of the samples in trial using Lasso results are shown in Table 2. The average number of extracted variables was 8.3 at a regularization strength of 0.03, and the maximum coefficient of determination was 0.269, which is the same as that of the monoaxial screw. In contrast, in the polyaxial screw, the number of explanatory variables extracted more than 1000 times was four, which is less than that of the monoaxial screw. Additionally, the maximum number of selected explanatory variables was lower (1455) than that for the monoaxial screw (2000). Thus, compared to monoaxial screws, polyaxial screws are less concentrated on certain explanatory variables and are more difficult to explain with a few distinct variables. The frequency range of the selected variables includes values greater than 10,000 Hz, which is not important for the monoaxial screw, indicating that the influence of the moving head extends over the entire analysis frequency range. This may be due to the fact that the vibration frequency spectrum obtained from the laser-induced vibration changed several explanatory variables owing to the influence of the head moving part of the polyaxial screw, resulting in fluctuations that appear as if they were random. However, as shown in the vibration frequency spectra in Figure 4 and Figure 5, it should be noted that the noise components are not completely random, as represented by white noise, and cannot be easily removed manually.

**Figure 10:**
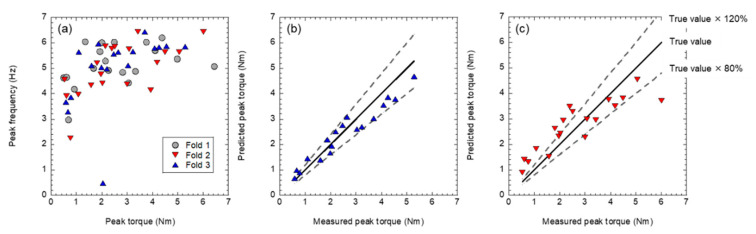
Demonstration of machine learning-based diagnosis of polyaxial pedicle screw evaluated by three-fold cross-validation: (**a**) fold term, (**b**) prediction result of Fold 3 with Fold 1 and Fold 2 training data, and (**c**) prediction result of Fold 2 with Fold 1 and Fold 3 training data.

We next discuss the results of Lasso in SVR. To verify the effectiveness of SVR, we performed cross-validation, in which we split the data to train and verify each other. Figure 10a shows the classification of the 56 plots in Figure 6 into three folds. Fold 1, including the minimum and maximum peak torque data, was used as training data to prevent the analysis of extrapolated data. For the other data, we sorted the data in the order of peak torque and prepared data sets with the *j*th, *j* + 1st, and *j* + 2nd data so that the peak torques contained in each fold are evenly distributed. Figure 10b shows the results of the cross-validation for Fold 3 data using Fold 1 and Fold 2 as training data. In contrast, Figure 10c shows Fold 2 data using Fold 1 and Fold 3 as training data. The SVR results were obtained using 30 variables extracted by Lasso, including a large number of variables with less than half the number (1000 times) of uses that are not shown in Table 2. In Figure 10b,c, the solid line indicates the correct value, that is, the measured value equals the predicted value, while the dashed line indicates the line of ±20% of the correct value. Figure 10b, which was validated with Fold 3, is the highlight of this study. The prediction accuracy was very high: 16 out of 18 data points (approximately 89%) fell within the ±20% range, with a coefficient of determination *R*^2^ of 0.816, a correlation coefficient of 0.964, and an error of the mean square of 0.00601. The coefficient of determination for this regression is comparable to the results of logarithmic fitting in a previous study on monoaxial pedicle screws [10], demonstrating that the same level of regression can be obtained for polyaxial pedicle screws. It is noteworthy that this prediction performance was achieved with a limited number of training data (38 points). In addition, even data showing a shallow peak frequency at a peak torque of approximately 2 Nm, as shown in Figure 10a, could be analyzed. The proposed diagnosis scheme can provide information on implant stability, even for measurement data that would be evaluated as false by conventional methods. In contrast, the accuracy of the Fold 2 data (Figure 10c) was lower than that for the Fold 3 data (Figure 10b), with a coefficient of determination *R*^2^ of 0.316, a correlation coefficient of 0.878, and an error of mean square of 0.0182. Because the dispersion of the Fold 2 data is larger than that of the Fold 1 and Fold 3 data, it is possible that when Fold 2 data are used for training data, a better SVR can be provided, but when the Fold 2 data are used for validation, the diversity brought by the variance is not reflected in training, resulting in deterioration of the validation results. The three-fold cross-validation revealed features that affect predictive accuracy depending on the training data characteristics of machine learning-based analysis. Currently, this machine learning-based diagnosis is a limitation with respect to usage in clinical situations and needs to be demonstrated in the future. However, in a previous study using a monoaxial screw, a good correlation between the implantation torque and L-RFA was obtained not only in the artificial bones but also in the cadaver bones [10]. This indicates that implant stability diagnosis using the vibration frequency spectrum obtained from L-RFA is also useful in a clinical situation. In clinical practice, the results in Figure 10 can be used as training data for Fold 1 to 3 data, as shown in the discarded cross-validation in Figure 9. Therefore, we can expect the performance as shown in Figure 10b.

In machine learning and deep learning, it is essential to use high-quality training data. As a limitation of the proposed diagnosis scheme, the implant stability evaluation shown in Figure 10b needs to be improved by accumulating more data. In particular, it is important to verify diagnostic performance when new conditions are added. For example, the location where the L-RFA is used is an important factor. Clinical and biological environments where various devices exist and noise is associated must be considered in the L-RFA analysis. In addition, it is necessary to optimize the analysis scheme for each type of implant because of natural frequencies and modes depending on the physical properties and shape, which are the measurement principles of L-RFA. However, a highly robust and usable diagnosis scheme can be obtained by accumulating and learning with a high-quality database in diverse conditions for L-RFA measurement. In this study, to compare with the conventional L-RFA analysis method, we performed machine learning-based analysis using only explanatory variables obtained from laser-induced vibration spectra and showed good analysis results. Furthermore, in clinical situations, the accuracy can be improved by clinical data, such as bone density, age, and gender, as explanatory variables.

## 4. Conclusions

In this study, we evaluated a polyaxial pedicle screw using L-RFA to reveal the influence of the movable head part on the vibration spectrum. We found that the laser-induced vibration frequency spectrum of the polyaxial screw was affected by the movement of the head. In such a situation, the spectrum obtained using the conventional method of correlation evaluation with a peak frequency in the spectral characteristics is difficult to analyze. Therefore, we developed a new machine learning-based diagnosis method. This diagnosis scheme divides the vibration frequency spectrum into multiple frequency ranges and extracts multiple spectral variables to prepare a large number of explanatory variables. A large number of explanatory variables were prioritized in terms of importance by Lasso, and highly accurate nonlinear regression analysis was performed even with a limited number of training data using SVR. The proposed machine learning-based diagnosis method demonstrated a highly accurate prediction of implant stability (peak torque) for polyaxial pedicle screws. Based on the achievement in the previous demonstration using artificial and cadaver bones with the analysis using only laser-induced vibrational peak frequency [10], the proposed machine learning-based analysis method has the potential to be used in clinical situations. Additionally, the experiments in this study were conducted using artificial bones covering human trabecular and cortical bones. The diagnosis scheme has the potential to be adapted not only for pedicle screws but also for other orthopedic implants and fasteners used in industry and architecture. We believe that the results of this study will prove to be an essential milestone in the practical application of L-RFA for implant stability diagnosis.

## Figures and Tables

**Table 1:** Result of explanatory variable selection by Lasso for monoaxial pedicle screw reported in the previous study [10]. These explanatory variables extracted more than 50% of the samples of trials.

Number of Selections	Frequency Range (Hz)	Explanatory Variable
2000	1000–5000	Peak frequency
1618	500–1000	Dispersion
1553	500–1000	Skewness
1510	150–500	Centroid frequency
1490	5000–10,000	Average power

**Table 2:** Results of explanatory variable selection by Lasso for polyaxial pedicle screws. These explanatory variables extracted more than 50% of the samples in the trial using Lasso.

Number of Selections	Frequency Range (Hz)	Explanatory Variable
1455	1500–6500	Peak frequency
1388	1500–6500	Centroid frequency
1344	0–25,000	Skewness
1290	0–25,000	Centroid frequency
